# Using mHealth to improve health care delivery in India: A qualitative examination of the perspectives of community health workers and beneficiaries

**DOI:** 10.1371/journal.pone.0227451

**Published:** 2020-01-15

**Authors:** Lakshmi Gopalakrishnan, Laura Buback, Lia Fernald, Dilys Walker, Nadia Diamond-Smith, in addition to The CAS Evaluation Consortium

**Affiliations:** 1 School of Public Health, University of California, Berkeley, United States of America; 2 Institute of Global Health Sciences, University of California, San Francisco, United States of America; 3 Department of Obstetrics and Gynaecology, University of California, San Francisco, United States of America; 4 Department of Epidemiology and Biostatistics, University of California, San Francisco, United States of America; ESIC Medical College & PGIMSR, INDIA

## Abstract

**Background:**

mHealth technologies are proliferating globally to address quality and timeliness of health care delivery by Community Health Workers (CHWs). This study aimed to examine CHW and beneficiaries’ perceptions of a new mHealth intervention (Common Application Software [CAS] for CHWs in India. The objectives of the study were to seek perspectives of CHWs and beneficiaries on the uptake of CAS, changes in CHW-beneficiary interactions since the introduction of CAS and potential barriers faced by CHWs in use of CAS. Further, important contextual factors related to CHW-beneficiary interface and dynamics that may have a bearing on CAS have been described.

**Methods:**

A qualitative study was conducted in two states of India (Bihar and Madhya Pradesh) from March-April 2018 with CHWs (n = 32) and beneficiaries (n = 55). All interviews were conducted and recorded in Hindi, transcribed and translated into English, and coded and thematically analysed using Dedoose.

**Findings:**

The mHealth intervention was acceptable to the CHWs who felt that CAS improved their status in the communities where they worked. Beneficiaries’ views were a mix of positive and negative perceptions. The divergent views between CHWs and beneficiaries surrounding the use and impact of CAS highlight an underlying mistrust, socio-cultural barriers in engagement, and technological barriers in implementation. All these contextual factors can influence the perception and uptake of CAS.

**Conclusions:**

mHealth interventions targeting CHWs and beneficiaries have the potential to improve performance of CHWs, reduce barriers to information and potentially change the behaviors of beneficiaries. While technology is an enabler for CHWs to improve their service delivery, it does not necessarily help overcome social and cultural barriers that impede CHW-beneficiary interactions to bring about improvements in knowledge and health behaviors. Future interventions for CHWs including mHealth interventions should examine contextual factors along with the acceptability, accessibility, and usability by beneficiaries and community members.

## Introduction

Community health workers (CHWs) are an integral component of the health workforce in low- and middle-income countries (LMICs), providing last mile services, especially in underserved areas [1]. Since the late 1970s and 1980s, CHWs have been a cornerstone of primary healthcare as envisioned by the Alma-Ata Declaration [2,3]. In the mid-2000s, CHW programs expanded based on the need to perform ‘task shifting’ from higher level medical providers, such as doctors and nurses, to lay workers, especially in response to the burden of the HIV epidemic. [4]. The adoption of Astana Declaration in 2018 reiterated the importance of primary health care and community health systems in improving population health [5]. CHW programs are considered to be part of long-term investment in strengthening community-oriented health systems and achieving universal health coverage (UHC) and health-related sustainable development goals (SDGs) in many LMICs [6].

CHWs form a crucial link between health systems and the communities they serve by improving access to and coverage of health services. This is supported by evidence demonstrating the effectiveness of CHWs in delivering a range of preventive, promotive, and curative services across a range of health domains [7–9]. However, CHWs often face challenges including maintenance of skills and knowledge needed for the complexity of tasks performed in the field, inadequate access to training materials, poor communication with their supervisors to handle complex situations, and difficulty in tracking home visits and follow-up appointments with their beneficiaries [10].

With the exponential rise of smart phone ownership and associated applications, digital health interventions are being used to support CHWs in their job through increased access to information, work planning, and decision support [10]. There has been a multitude of digital health interventions for CHWs in LMICs that cut across the 12 functions defined in the mHealth and Information and Communication Technology framework for Reproductive, Maternal, Neonatal and Child Health (RMNCH). This includes client education and behavior change communication (BCC), sensors and point-of-care diagnostics, vital registration, data collection and reporting, electronic health records, electronic decision support, provider-to-provider communication, provider work planning and scheduling, provider training and education, human resource management, supply chain management, and financial transactions and incentives [11].

These digital health tools are often designed to aid the CHWs to perform their services better. Further, these tools may also incorporate beneficiary-facing content intended to improve beneficiaries’ knowledge, and/or practices. Though many feasibility studies and evaluations have been done to test the effectiveness of these tools, few studies have documented perspectives of both CHWs and beneficiaries on the digital health tools.

Our literature review suggests encouraging evidence of acceptance of digital health tools based on CHW perspectives. The range of tools for CHWs involved in these studies include job aids, scheduling systems, checklists and/or client education and behavior change communication tools. However, of the eight studies conducted in both Asia and sub-Saharan Africa, only two of them sought beneficiary perceptions of the interventions and how it influenced the uptake of services among beneficiaries [12–19]. In studies where beneficiaries’ perspectives were sought, they reported better quality of interactions with CHWs, active participation in care-seeking, and better quality of services [14,16].

This sparse evidence on beneficiary perspectives and acceptability of these interventions presents an important research gap in the field of digital health interventions for CHWs. Seeking beneficiaries’ perceptions might bring to fore socio-cultural contextual barriers that impede the uptake of the digital health interventions and explain plausible reasons for the failure of these interventions in translating into health outcomes.

Furthermore, recent systematic reviews conducted on mHealth targeting CHWs to improve RMNCH in low-resource settings find that most studies are observational, are small-scale or pilots, and are methodologically weak, with very few experimental studies conducted to date [20–22]. These reasons coupled with heterogeneity of interventions and outcome measures, and length of evaluations being generally short, are some plausible reasons that studies have not been able to determine if mHealth interventions can impact maternal and neonatal health outcomes. Further, there is a need for robust evidence to demonstrate the impact of mHealth efforts on the health interaction itself (between CHW and beneficiary), and the views of beneficiaries more broadly.

### Beneficiary -CHW-health system framework

Scott et al. developed a framework of the CHW-health system based on a review of 122 articles specific to the Indian CHW program [23]. The framework is situated within the context of the larger health system, as most publications have been authored from a health systems perspective. The center of the framework is focused on program outcomes, such as CHW performance, knowledge, and motivation. The majority of publications (79 of 122) include content on CHW performance and program inputs, focused on training and capacity building (69 articles). This program input-outcome approach is most common in the CHW literature, including for mHealth related literature, as mHealth is a program input for CHWs designed to improve their service delivery, program outcomes and impact. However, the framework demonstrates that both the health system and community factors overlap with the CHW program to influence the relationship of program inputs and outcomes.

As CHWs are selected from the local community to serve their own community and act as a link between the formal health system and the community, the extent of embeddedness in the community can define the level of CHW acceptance and investment. The embeddedness can also influence the level of the CHW engagement, including building of trust and empathetic relationships between the CHW and community members [24–26]. In communities with a functional CHW-community interface, the CHWs are recognized and share high levels of trust and stronger relationships with community members, CHWs are well positioned to conduct home visits and counsel beneficiaries on behavior change topics, enabling them to deliver program objectives.

**Fig 1:**
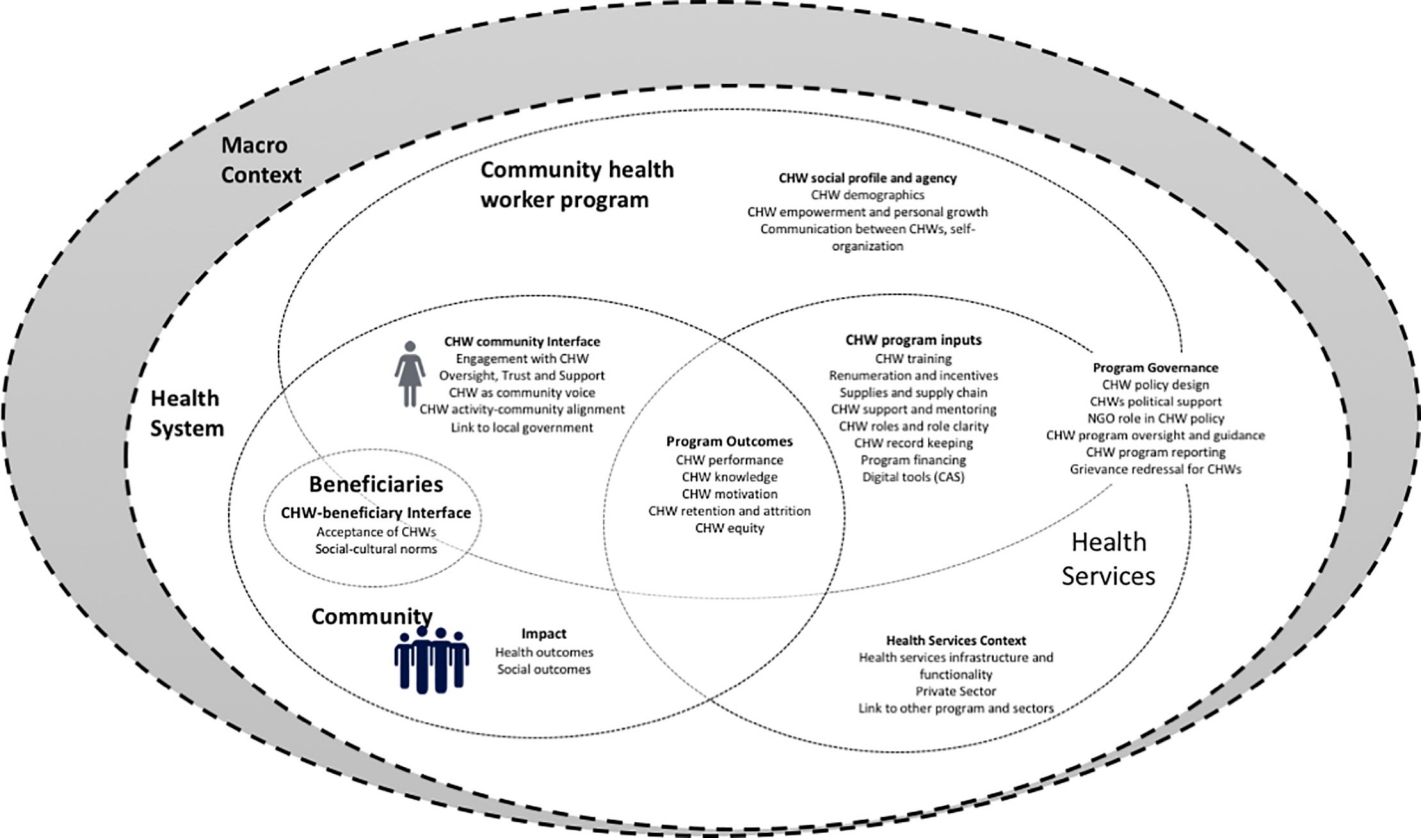
Framework depicting beneficiary-CHW-health system interface[23].

Scott et al. show that topics of CHW-community interface and CHW social profile, agency and community embeddedness are less commonly addressed in the literature. For example, within the CHW-community interface, 34 articles addressed community engagement with CHWs, and within CHW profile/agency, only 14 articles addressed CHW empowerment and personal growth. In this study, we adapted this framework as depicted in Fig 1 to incorporate the beneficiary perspectives, including beneficiary acceptance of the CHW and their use of CAS for interactions, perceptions of CHW communication, and community-embeddedness and trust [23].

### The Integrated Child Development Services (ICDS) program

In India, health and nutrition interventions are delivered via a network of CHWs through two flagship programs–the National Health Mission (NHM) and the Integrated Child Development Services (ICDS). The CHWs responsible for service delivery at the community-level include Accredited Social Health Activists (ASHAs) of the National Health Mission and the Anganwadi Workers (AWWs) of the Integrated Child Development Services. AWWs are part-time female CHWs who receive an average monthly fixed honorarium of about USD 60 (INR 4500) for the services they provide, though the honorarium varies by state. A typical AWW is from the local village (for the remainder of this paper we will refer to CHWs in this context as AWWs), with at least ten years of schooling and an age at recruitment between 18–35 years. AWWs deliver services to pregnant and lactating women and children below the age of six years through Anganwadi Centres (AWCs), serving a catchment area of a population of 1,000 [27–29]. Under the ICDS program, AWWs provide monthly food supplements, facilitate health check-ups, conduct pre-school education for children 3–6 years of age, and counselling on pregnancy care, infant and young child feeding practices both at the AWC as well as during home visits to beneficiaries. They also work closely the ASHA workers in their villages to plan and organize a monthly fixed-day event, Village Health and Nutrition Days (VHND) for immunization and shared health services.

Few observational studies on ICDS have found an association between ICDS service delivery and improved maternal nutrition and modest improvements in child nutrition[30–32]. However, past evaluations of the ICDS program have identified many gaps including inadequate infrastructure, low service delivery by CHWs, insufficient training, limited supervision, and irregularities in record keeping, among others [33–35]. Further, the nutritional indicators from the most recent Demographic Health Surveys of India highlights low coverage levels of ICDS. For example, only about 59% of the children under the age of 6 years received any service from an AWC, a little over 50% received food supplements, and 47% were weighed [36].

In 2012, the Government of India, with support from the World Bank, launched the ICDS system including the ICDS Systems Strengthening and Nutrition Improvement Program (ISSNIP) focused on an upgrading of the infrastructure and capacity building of CHWs to build their content knowledge on health and nutrition. Around the same time in 2013, a pilot of an mHealth intervention was implemented in a district in Bihar to improve ICDS service delivery that provided smartphone applications to both sets of CHWs, the AWWs and the ASHA workers. A randomized controlled trial of this intervention found a significant increase in the proportion of beneficiaries receiving visits from AWWs at different life-stages and also some improved practices related to maternal and newborn health [37].

At the national level, CAS was conceptualized at the Ministry of Woman and Child Development (MWCD) to create an information-technology enabled solution to improve the ICDS program. Subsequently, an at-scale mHealth intervention, the Common Application Software (CAS) for the AWWs was rolled out in 162 high malnutrition burden districts across eight states in 2015. The remaining scale-up of CAS to the remaining AWCs nationwide is currently ongoing in a phased manner under the National Nutrition Mission launched in 2018. As of September 2019, CAS had been rolled out in 11 different languages to 376, 537 AWWs across 213 districts in 24 states in India and had a reach of about 34.4 million beneficiaries including children and pregnant women and lactating mothers.

### The mHealth intervention, Common Application Software (CAS)

The mHealth intervention, CAS is comprised of three components:

- The AWW CAS App (referred to as CAS hereafter): CAS was developed on an open-source mobile platform (CommCare) and digitizes ten of the eleven paper registers maintained by the AWWs, it also acts as a job aid for the AWWs to improve their service delivery. CAS contains eight modules that enable the AWWs to track beneficiaries in their catchment area by their names. It supports their daily activities including recording disbursement of food supplements to beneficiaries, maintaining daily record of supplementary nutrition provided, and attendance and pre-school education of children at the AWC. It also improves record-keeping, enables AWWs to accurately plot of growth status of children, allows AWWs to track pregnant women and children due for immunization, prompts AWWs to conduct home visits at critical life stage of the beneficiaries and enables use of standardized checklists and BCC videos to be used during home visits. There are four BCC videos on family planning, birth preparedness, complementary feeding, and sanitation embedded as part of the checklist and part of the video library as well in the regional language of the state. For instance, in Bihar and MP, the videos are played in Hindi.
- The Lady Supervisor CAS App: A lady supervisor manages and supervises a group of approximately 20 AWCs and their version of the app is installed on smartphones or tablets provided by the Government. This App is designed to help the supervisors prioritize AWCs that are not adequately up to 6 years, especially in the 0–3 years age group performing and providing support for those specific CHWs.
- Dashboards: The raw data fed through the CAS application filled by the AWWs and supervisors is aggregated to the system to allow for real-time monitoring via web-enabled multi-level dashboards at the block, district, state and the national-level. This real-time monitoring and tracking nutrition and growth indicators for pregnant and lactating mothers, and children is expected to ensure data-driven decision making for policy and implementation.

Smartphones with Android operating system are pre-loaded with the CAS application. These phones are then given to AWWs and a detailed training is provided on the use of smartphones and CAS. Further, helpdesks at sub-districts and district levels are established for ongoing technology-support [38].

Our theory of change for the CAS mHealth intervention is that CAS serves as a job aid for AWWs to improve home visits through automatic reminders for home visits based on life-stage of the beneficiary, alerts for approaching or missed home visits, and checklist and videos to be used during home visits to engage with the beneficiaries. Overall, through use of CAS, we expect that AWWs will be able to better prioritize their home visits both in terms of timeliness, frequency of visits, and improve quality of counselling through use of standardized checklists and videos. This is expected to result in higher knowledge of beneficiaries and perhaps, better pregnancy care, birth preparedness, family planning, and infant and child feeding practices by the beneficiaries.

Our team is also wrapping up a parallel CAS impact and process evaluation in two states of India, Madhya Pradesh (MP) and Bihar; details of sampling for the baseline survey of the impact evaluation are available in our protocol paper [38] and the results will be published separately. These two states were chosen for the evaluation because of the ability to select another ISSNIP district as a control district, willingness of the states to support the evaluation and based on suggestions by the MWCD and the funding agency, the Bill and Melinda Gates Foundation.

### The MP and Bihar context

According to the 2015–16 Demographic Health Survey, Madhya Pradesh and Bihar have a high burden of under-five mortality with 65 and 58 per 1000 live births respectively. The stunting levels for children below the age of five are high at 43.6% and 49.3% in MP and Bihar respectively, and anaemia of 54.6% and 58.3% among pregnant women in MP and Bihar respectively. Educational attainment of women in MP and Bihar is quite low with only 14% and 12% of women aged 15–49 respectively having completed 12 or more years of schooling. In Bihar, the coverage indicators of ICDS are low. Among children below the age of 6 years, 49% received any service from an AWC and about half of the mothers of children who were weighed at an AWC received counselling from a AWW. 35% of mothers with children below the age of 6 years received any service from an AWC during pregnancy and lactation. Madhya Pradesh is better in terms of coverage of ICDS services with 63% children below the age of 6 years receiving any service from an AWC and 62% of mothers who were weighed at an AWC received counselling from a AWW. Further, 71% of mothers with a child below the age of 6 years received any service from an AWC during pregnancy, and 66% received any service during lactation [36].

In this qualitative study, our goal was to seek perspectives of AWWs and beneficiaries on the uptake of CAS, how CAS may or may not have changed the quality and nature of interactions during the early adoption of CAS, and barriers impeding use of CAS during service delivery. Further, we also shed light on the important underlying contextual factors related to AWW-beneficiary interface that might have a bearing on the uptake of CAS using the framework described above.

## Methods

### Sampling

This qualitative study was conducted in the intervention areas of Ujjain district of Madhya Pradesh and Samastipur district of Bihar in March-April 2018. AWWs were purposively sampled for in-depth interviews from the baseline survey sample. Based on the baseline survey sample list of AWCs, a list of eligible of AWWs were selected to be interviewed if they had been using CAS for at least three months prior to the interview. An effort was made to get a spread of educational attainment and age of AWWs to understand the variation in adoption and usage of CAS. In the catchment area served by each AWW interviewed for the study, a maximum of two beneficiaries, including one pregnant woman and one lactating woman with a child 0–24 months, were selected. The objective was to find beneficiaries who were registered and receiving services from their local AWC and had received some exposure to CAS–either had seen their AWW using CAS in front of them or had at least heard of it. However, given that the intervention was in its initial stages of rollout (*within 6 months of training on CAS for AWWs*), some beneficiaries interviewed had never heard of CAS or had not received any exposure to the technology. Additional respondents were interviewed until the study team collectively agreed that data saturation was achieved.

### Interview guides

The overarching goal of the interviews with AWWs was to seek perceptions of use of CAS, changes in service delivery and interactions with the beneficiaries after introduction of CAS, and challenges faced by the AWWs in use of the CAS. The interview guides for beneficiaries focused on perspectives of their interactions with their AWWs, early exposure to CAS, specifically the videos since they were an integral behavior change communication tool within CAS. The interview guides were piloted in the field for two days before finalizing.

### Data collection

Face-to-face, in-depth interviews were conducted in Hindi by a team of four Indian female researchers (including the first author (LG)) with strong background in qualitative methods and a minimum of 16 years of formal education. Saturation was achieved with a total of thirty-two AWWs and fifty-five beneficiaries interviewed [39]. None of the interviewers worked in a community-setting with AWWs or had previously met any of the participants. All participants were recruited through the registers maintained by the AWWs. All of the interviews were audio-recorded after seeking informed consent from the participants for the interview and recording specifically. The average length of interviews was 90 minutes for AWWs and 30 minutes for beneficiaries. Interviews were conducted in a private setting either at the homes of the beneficiaries or at the Anganwadi centres for the AWWs.

### Data analysis

All the audio-recorded interviews were anonymized, transcribed verbatim and translated into English by an external qualitative research firm. Thematic analysis was chosen as the method to analyse the data from the interviews because it is useful for applied research with an emphasis on policy and practice. Further, it also offers a systematic framework to code qualitative data in order to identify patterns across the data. We followed the steps involved in thematic analysis, including familiarizing ourselves with the data, generating initial codes, searching, reviewing, and finally defining themes [40].

A team of four female researchers (two in India, two in the US including the first author, LG) reviewed and coded the transcripts in Dedoose using a thematic analysis theory approach [41]. Additional codes were developed through the coding process in an iterative manner. At least 10% of the AWW and beneficiary transcripts were double-coded by all the researchers to ensure reliability in coding. A thematic analysis was conducted by the first author (LG), the senior author (NDS) and a research assistant to develop themes based on the code reports generated.

### Informed consent

Informed consent was obtained from all participants before any interviews were conducted.

Study protocols have been reviewed and approved by institutional review boards at the University of California, Berkeley (Ref. No. 2016-08-9092), and the India-based Suraksha Independent Ethics Committee (Protocol No. 2016-08-9092). The trial is registered with the ISRCTN registry (https://doi.org/10.1186/ISRCTN83902145).

## Results

AWWs in Madhya Pradesh were an average of 45 years old and those in Bihar were 38. They had many years of experience working as a Community Health Worker, had the equivalent of a high school education, were Hindu, and were likely to own a smart phone (Table 1).

**Table 1:** Characteristics of AWWs and beneficiaries in Bihar and Madhya Pradesh.

	Bihar	Madhya Pradesh
**AWWs**		
Total number of AWWs interviewed	15	17
Average age of AWWs in years	38.2±6.8	44.7±8.9
Average years of schooling of AWWs	13.2	11.2
Religion of AWWs	Hindu (15/15)	Hindu (16/17)
Average number of years as a AWW	12.8	18.5
First-time smartphone ownership	100%	80%
**Beneficiaries**		
Total number of beneficiaries interviewed	25 (13 lactating women, 12 pregnant women)	30 (17 lactating women, 13 pregnant women)
Average age of beneficiaries in years	24	23
Average years of schooling	4	5
Religion	Hindu (25/25)	Hindu (27/30)
Average age at marriage in years	17	18
Below Poverty Line (BPL) households	64%	63%
Caste	16% Other Backward Classes, 48% Scheduled Caste, 24% General Caste	50% Other Backward Classes, 23% Scheduled Caste, 7% General Caste
Average birth parity	2.4	1.4

The findings are broadly described based on relevant aspects of the beneficiary-CHW-health system interface framework presented earlier.

### 1. Role of CAS in influencing AWWs’ community embeddedness

Overall, our findings from interviews with AWWs suggest that where AWWs had limited levels of community interface and embeddedness, CAS provided an enabling environment for achieving program outputs by legitimizing AWW’s counselling messages and improving her engagement with the community. AWWs perceived CAS to be a better medium for communicating with beneficiaries than in-person verbal communication because beneficiaries were more likely to pay attention to and value the CAS.

*“Initially I used to give information verbally*, *so people didn’t listen to me*. *They used to say that CHW keep talking like that all the time*. *They didn’t find it important to listen to me*, *but now it is different with the mobile*, *I can show them videos or click pictures on it*. *So now when people ask me what you are doing in this phone*, *then I show everything in the mobile*.*” (AWW*, *Madhya Pradesh*, *Age 52*, *8*^*th*^ *grade education****)***

As evident in the quotes, some AWWs seemed to feel that beneficiaries were disrespectful of their time and devalued the information being provided before CAS, even using terms like “taunting” to describe the tone of the interactions. One AWW strongly felt that the phone legitimized her work and CAS had made a substantive difference in the quality of interactions, with beneficiaries showing interest in them and appreciating them.

*“Now I am able to show them that I work*. *Earlier they used to taunt me by saying that we*, *the AWW*, *don’t do anything*. *So now I am able to show them that I have this mobile from government to do my work…*. .*it has made a difference*. *Earlier they didn't use to come to the centre even on Tuesdays fixed day service*; *used to be very difficult to have them gather here*. *But now they come over just after once my (helper) informs them to come to the centre*. *Now all females come over…*..*Because they are able to appreciate my work now*.*” (AWW*, *Madhya Pradesh*, *Age 34*, *8*^*th*^ *grade education)*

With CAS installed on smartphones, the AWWs were able to use features of the phone and the App to upload pictures of children eating hot cooked meals and community events including baby showers, or children’s birthdays at the Anganwadi Centre. They also reported showing videos on family planning, birth preparedness, complementary feeding to beneficiaries during their home visits and at the Anganwadi Centre. These features were perceived as helping the AWWs engage beyond their direct beneficiaries, pregnant and lactating women, and gain acceptance of important decision-makers in the household, including mothers-in-law. In few instances, AWWs described building trust with mothers-in-law as being critical in increasing their daughters-in-law’s ability to interact with the AWW and visit the AWC, and to ultimately receive the information and care they needed. This was particularly true in case of beneficiaries with low education who could not absorb all of the information in one session and could not report back to their mothers-in-law, as described below:

*“Previously what happen was either mother-in-law or daughter-in-law used to come to the Anganwadi Centre*. *The women who were educated share all the information with other family members on returning back home and some of them forget what they have heard at the Anganwadi Centre*. *In such situations*, *the mother-in-law asks the daughter-in-law the reason of going to the Anganwadi Centre*. *Then she tells the mother-in-law that AWW had called me there*. *The next day the mother-in-law comes to me asking why I called her daughter-in-law to the Anganwadi Centre*. *Then I show her the pictures and videos of the event which I recorded*. *I show them the number of women who attended the event and the songs and dance they performed*. *Then next time the mother-in-law trusts me and tells the daughter-in-law to go the Anganwadi Centre*.*” (AWW*, *Madhya Pradesh*, *Age 52*, *8*^*th*^ *grade education)*

In addition to improving the level of engagement, credibility and embeddedness in the community, AWWs perceived that CAS also changed women’s perceptions of the government and the work that the government was doing to try to provide information and services.

*“They say that the government is doing good work*. *They understand better when they see it*. *It is not so clear when only I speak*. *They like watching the video (AWW*, *Bihar*, *Age 32*, *Post-graduate education)*

While AWWs perceived CAS as being an important facilitator in improving their own credibility, engagement with beneficiaries and their community embeddedness, beneficiaries had more nuanced views. Of the very few beneficiaries who did note a change since the introduction of CAS, they did show evidence that their trust in the information itself had increased, because it came from a mobile phone, as opposed to from the AWW herself. As explained by one of the women in the quote below, she trusted the mobile phone more than the AWW.

*“I used to ask AWW that why she keeps this mobile with her then she said that she has all information in that*, *about us and this is her duty to tell us everything and to take us for tests and check-ups and to provide beneficial information to us …*.*If she tell us orally then we won’t believe but we have to believe when she show video in mobile then we do what she told”* (Mother, Madhya Pradesh, Age 26, 5^th^ grade education)

However, the majority of beneficiaries felt that CAS had not improved their relationship with AWWs or how they viewed their AWWs. One potential reason for this might be that the CAS had been operational for approximately 6 months or less by the time of this study and many beneficiaries had not seen the AWW using the CAS in front of them or had not seen the videos. Of the handful of beneficiaries who had seen the video, they were ambivalent about receiving counselling either from a AWW or through animated videos. This was often related to use of the local language which some beneficiaries felt CHWs were better able to communicate in, while others felt that the video’s language was easier to understand.

### 2. Role of CAS in changing beneficiary-AWW interactions

In addition to influencing AWWs role in the community, findings suggest that CAS also had an effect on interactions and provision of information between AWWs and beneficiaries.

#### Positively accepted tool for health information provision with beneficiaries

AWWs mentioned that CAS piqued beneficiaries’ interest in the information that AWWs offered during their counseling sessions. They also attested that it provided a platform for other women in the neighborhood to gather around the AWW and receive information. Based on the self-report by AWWs, a majority of AWWs felt videos played a crucial role in engaging women.

*“These days women ask me to show them the video*. *Once a woman starts watching the video*, *women from her neighbors will also join her*. *They come and ask what I am showing her on mobile*. *So*, *I ask them to come and watch the video that is being played on the mobile*.” *(AWW*, *Madhya Pradesh*, *Age 52 years*, *8*^*th*^ *grade education)*

AWWs felt that CAS not only stimulated the beneficiaries’ interest in seeking information and increased engagement with beneficiaries, it also motivated a change in care seeking behaviours for a few beneficiaries. An illustrative quote below from an AWW who felt that beneficiaries were more motivated to seek information and care:

*“Due to this (CAS) our village has improved*, *our department has improved*. *Counseling mothers during the home visit has shown the greatest improvement in changing their behavior*, *for instance*, *many mothers would not agree to give vaccinations to their children*, *not even polio drops*. *But after so much counseling and motivation their behavior has changed*. *Now they have become so aware that they come to me on their own and bother me each time and ask when is the next vaccination day going to be*?” *(AWW*, *Bihar*, *Age 38 years*, *10*^*th*^ *grade education)*

#### AWW-community and beneficiary alignment

In certain interviews, contrary to the lack of trust highlighted earlier, a few beneficiaries felt that AWWs gave them useful information and counselled effectively without specifically referring to CAS. This highlights that the beneficiaries were highly engaged with their AWWs and trusted their AWWs, even without the use of CAS. The underlying nature of the AWW-beneficiary relationship influenced the extent to which CAS enhanced the beneficiary-CHW interface.

Some felt that they trusted the CHW, even more than doctors, and, as one beneficiary described: *“we discuss our problem with her and follow her instruction as she is the only one on which I can rely on*.*” (Mother*, *Bihar*, *Age 21*, *12*^*th*^ *grade education)*.

Another beneficiary described that she specifically sought out the CHW because she knew they would provide high quality information: *“I go to her home as I know she will show me good things and give me important information” (Mother*, *Madhya Pradesh*, *Age 26*, *5th grade education)*.

#### Influence of CAS on the quality of interaction with beneficiaries

AWWs overwhelmingly discussed how CAS had made their lives easier. The home visit scheduler feature of CAS enabled visiting a specific beneficiary at the appropriate time depending on the life-stage of the beneficiary, information provision including pregnancy care, birth preparedness, new-born care through use of checklists during home visits. CAS also provided the convenience of entering data into the phone without needing to repeat entries. However, as can be seen below, what might be beneficial for the AWW (shorter visits), might actually not be ideal in terms of the quality of the interaction in the home of the beneficiary.

*“Now in a day I am able to complete 2 home visits*. *I spend approx*. *10 minutes in each home and it's done*! *I go and take a round of the center and I am able to provide information to the beneficiaries visiting the centre using this phone*. *It has made my work very convenient” (AWW*, *Bihar*, *Age 38 years*, *10*^*th*^ *grade education)*

Again, conversations with the AWW revealed the flip side of convenience and desirability of the video, which, as discussed above, both AWWs and the women themselves liked. As illustrated by the two quotes, AWWs reported actually not having to, or potentially choosing not to have a dialogue and counsel the beneficiaries because they can use the videos.

*“It is more convenient today [to do home visit] as we don’t have to speak much*, *we open the video and make them listen*.*” (AWW*, *Bihar*, *Age 42 years*, *12*^*th*^ *grade education)*

*“The video saves time; it is understandable*, *convenient*, *people understand it easily too*, *they don’t understand when everything is explained on paper*. *But they understand everything when they listen to it*. *I even don’t have to speak…*. *I show the video and she watches it*. *It speaks in local language so she understands it too*.*” (AWW*, *Bihar*, *Age 32 years*, *Post-graduate education)*

#### Fidelity of CAS intervention: Mixed views of beneficiaries on the role of CAS in changing beneficiary-AWW interface

Interpersonal communication is a key component of behavior change intervention design, as the interpersonal realm is a key component of the five-component socio-ecological framework for behavior [42]. CAS is intended to be a job aid to enable AWWs to harness interpersonal communication for behavior change, but there were mixed reports on the fidelity of this intervention. Some beneficiaries report their perception of good communication approaches, while others report less personal, rushed communication methods. One woman felt that the AWW only visited her home when she was being watched by supervisors (“Madam”).

*“Interviewer*: *Ok so how is your relation/ terms with Anganwadi worker (AWW)*?

*Respondent*: *She talks nicely but never visited my house until or unless madam does not tell her or when madam with her then only she comes*.

*Interviewer*: *Ok*

*Respondent*: *When other officers visit our home then only she comes along with them otherwise not” (Mother*, *Bihar*, *Age 28*, *no schooling)*

Beneficiaries corroborated the AWWs’ descriptions of short visits, noting that the AWW usually stayed for short periods of time and did not focus deeply on education and counseling.

*“She does not stay for long time*, *she only stays for 1–2 minute*. *She usually come for giving polio drops or for calling children for immunization” (Mother*, *Madhya Pradesh*, *Age 24*,*12*^*th*^ *grade education)*

*“I don’t know about the time but she stays for a little while…5 to 10 minutes*, *she stays for 5 minutes only…Meaning she is also in a hurry and sometimes says that she has to go here or there*.*” (Mother*, *Madhya Pradesh*, *Age 21*, *8*^*th*^ *grade education)*

While some women reported seeing a video, this number was fairly low. Many women in our study reported not having seen a video, although those that mentioned verbal interactions with AWWs felt that the information given by the AWW was accessible to them.

*“Interviewer*: *Did she ever show you any video regarding what types of food should be given to the child*?

*Respondent*: *Whatever she tells the same type of food I give to the child*.

*Interviewer*: *She has never shown you any video*?

*Respondent*: *No*, *never*.*” (Mother*, *Bihar*, *Age 30*, *5*^*th*^ *grade education)*

*“She doesn’t show me the video but whatever information she gives me verbally that is also easy to understand*.*” (Pregnant woman*, *Bihar*, *Age 22*, *No schooling)*

Others reported seeing the video, but do not clearly remember the content, which suggests the mother may not give full attention or the video may be difficult to understand.

*“Respondent- I have seen videos before and after my delivery*. *I think I have watched videos 3–4 times*.

*Interviewer*- *Don’t you remember what was in that video*?

*Respondent*- *There was a doctor who was telling about diet and health of the children*. *I actually don’t remember the full video*.*” (Mother*, *Madhya Pradesh*, *Age 22*, *5*^*th*^ *grade education)*

Such perceptions are important to consider, given the objective of the video to improve communication and change behaviors. CAS or mHealth interventions do not alone guarantee the fidelity of program design.

### 3. Diverging viewpoints between AWWs and beneficiaries

Findings from the data revealed a discrepancy between the AWWs’ self-report about interactions with beneficiaries and the self-reports from the beneficiaries themselves who mostly said they had not interacted with AWWs. Given this apparent discrepancy, we probed AWWs and beneficiaries about this disconnect. Both AWWs and beneficiaries acknowledge lack of availability of the beneficiaries and mistrust of AWW as important factors driving this disconnect. They are described in detail below.

#### Physical limitations/unavailability of beneficiaries during home visits

Some AWWs reported difficulties in connecting with beneficiaries because they worked as day laborers or engaged in agricultural field work and thus were not at home when the AWW visited. AWWs explained how, in these cases, they would have to wait for beneficiaries or return at another time to meet with them. Hence, many AWWs explained that they would instead counsel another family member, assuming that these other household members (mothers-in-law, other daughters-in-law) could provide that information to the intended recipient.

*“This is the harvest season*, *[the women] go out to work in the fields and [do not] come back [until the] evening*. *Her mother-in-law is there… so we explain to her*.*” (AWW*, *Madhya Pradesh*, *36 years*, *12*^*th*^ *grade)*

Other AWWs felt that the beneficiaries were not interested in receiving information from AWWs and thus made other household members, such as mothers-in-law, listen to the AWW.

*“They say that they have some other work to do and they don’t have time so I should let them go*. *What do I do*, *they make their mother-in-law sit and leave*. *Some part of the discussion they listen to and rest of it has to be explained to her mother in law (laughing) I have to do my duty*.*” (AWW*, *Bihar*, *32 years*, *Undergraduate education)*

Beneficiaries corroborated some of these experiences, explaining that they have a lot of work or live a long distance from the centers, which, in some cases meant that they did not even recognize the AWW when they saw her. Beneficiaries also reported lack of time, interest, and difficulty in understanding the content provided by the AWW or the AWW herself:

*Interviewer*: *Do you meet her and listen to what she tells you or stay busy with some other work*?

*Respondent*: *Yes*, *I meet her sometimes*, *and sometimes it is not possible as I stay busy in my work*.

*Interviewer*: *Ok*, *had she shown you any video when she comes for the home visit*?

*Respondent*: *If she comes then she will obviously show me the video*.

*Interviewer*: *What was shown in the video*?

*Respondent*:*(again laughing) I don’t remember (Mother*, *Bihar*, *24 years*, *No schooling)*

AWWs also explained how socio-cultural and gender norms prevent young newly married women from stepping out of their home or to be seen in public, and thus seclusion of daughters-in-law within the household also limited the AWWs ability to interact with the beneficiaries. It should be noted that this should not be as big of a factor for home visits, and, theoretically, would only apply to visits to the AWC. In certain cases, there are cultural norms around leaving the home after giving birth to a child that prevent women who recently delivered from accessing services at the AWC, such as not being permitted to leave the home until over a month after delivery.

*“Maybe they were away and they did not see*. *Their mothers in law must have seen or the daughters in law were not allowed to come out so that is why they said so…*. *The village people are such they do not allow daughters in law to come at front…*. *Yes some daughters in law come*, *but some are not allowed by their mother in law*.*” (AWW*, *Madhya Pradesh*, *Age 53*, *10*^*th*^ *grade education)*

*“If in case of Rajput community*, *they do not send brides out*. *Tell to their household members whatever you want to convey*. *They let do but only when there is no one at home or only female members are at home*. *They do not let them come out as if they would go out somewhere…*. *I go to such place sometimes and explain and tell them*. *Rest is their wish*, *we can’t force them*.*” (AWW*, *Madhya Pradesh*, *Age 24*, *12th grade education)*.

*It is possible that [the beneficiary] may not have been shown the video and the information is given verbally without showing the video in the mobile*. *[It] also happens that [the mother in law comes out of the house to meet [the anganwadi worker] so [she has] to give the information to her or sometimes the women [the anganwadi workers] want to meet are busy with household chores then they don’t come out to meet [her so they just talk to the mother in law and return back]*. *[The new mothers come to the AWC*, *and they are allowed to come out of the house after delivery] after one and a half months*. *(AWW*, *Madhya Pradesh*, *Age 54*, *Graduate)*

Another commonly discussed barrier to interactions with women was the practice of pregnant women returning to their natal homes or parent’s homes during pregnancy, for delivery, and through some of the postpartum period. While some AWWs checked on these women via the phone or through family members, many AWWs were not able to communicate with and give information to women in that time period.

*“Interviewer*: *So do you even do house visits of the daughters or not*?

*Respondent*: *No I don’t go for home visits*, *another type of community health worker goes to their house for the delivery and when immunization takes place they ask me to make entry in the due list so I use to do it*.*” (AWW*, *Bihar*, *Age 32*, *undergraduate education)*

Women also discussed the return to their natal home as a barrier.

*“Interviewer*: *Ok so when she used to come*, *for how long she used to stay here at your home*?

*Respondent*: *10–15 minutes*

*Interviewer*: *She stays here for 10–15 minutes*. *Did you meet her*? *What did she tell you*?

*Respondent*: *Yes*

*Interviewer*: *What did she tell you*?

*Respondent*: *If I was here*, *I met her*. *If I was at my parental home*, *then couldn’t meet*.*”*

*(Mother*, *Bihar*, *Age 21*, *12*^*th*^ *grade education)*

#### Low level of trust between AWWs and beneficiaries

In direct contrast to the sentiments expressed above about CAS improving trust with beneficiaries and community members, when probed about why beneficiaries reported not seeing the mHealth intervention or video, many AWWs expressed deep, underlying mistrust and even a sense of maliciousness on the part of beneficiaries.

*“No madam we show them*, *show them here also*, *sometimes when we go to the home visit then we show them there also*. *There are many who went somewhere or who is not present there so how will we show to them like went to her parents’ house*, *is not here on that time so how will we show it to them*. *The work that we are doing today this is the same*. *Look if four women is there and 3 are saying good then one will be saying bad things for me also*, *she does not want us to work properly*.*” (AWW*, *Madhya Pradesh*, *Age 46*, *5*^*th*^ *grade education)*

As can be seen in the quote below, AWWs are uncertain if beneficiaries are listening but forgetting, or if beneficiaries are lying outright.

*“Many of them lie…*. *Many reasons are there*, *like they don’t pay attention when AWW tells them*. *The conversation gets over and when an external person comes they say that they don’t know anything though they had seen and heard it*.*” (AWW*, *Bihar*, *32 years*, *Undergraduate education)*

Regardless of the reasons for the contradictory reports, it is clear some AWWs feel mistrust and disconnect with the community as well as disrespect from some of the beneficiaries they serve in the community. It is not possible to expect an mHealth intervention to fix or function smoothly in light of these underlying AWW-beneficiary relationship dynamics.

### 4. Technological barriers to AWWs’ uptake of CAS

In addition to the aforementioned challenges with AWW-beneficiary relationship, there were also some technical barriers that impeded AWWs with showing the videos and using the phone in front of the beneficiaries. AWWs in both states reported facing technical challenges with their phones including the phone heating up, or phone freezing while entering data that hindered their service delivery.

“*Like during immunization program*, *if it stops working suddenly or gets hung or if there is any network failure*, *then I wait for a day or two*. *If still it’s not working*, *I show it to Sir (help desk official) or during meetings if I get time*. *The details which are important*, *I have to update timely*, *so I have to get it repaired immediately*. *“(AWW*, *MP*, *52 years*, *8*^*th*^ *grade education)*

A large majority of AWWs also faced issues with network, internet when it came to synchronizing the data with the servers. Further, nearly all AWWs did not have an electricity source in their AWC to charge their phones.

“*The data in this hardly works*, *madam*, *so have to connect with some other phone network*. *Very slow*, *it works in the evening or after four days*, *sometimes the forms are all bunched together*.*” (AWW*, *MP*, *45 years*, *Undergraduate education)*

*“We don’t have electricity in our AWC*, *it gets discharged (battery runs out) that’s why I keep my phone off*.*” (AWW*, *MP*, *44 years*, *12*^*th*^ *grade education)*

## Discussion

Our qualitative interviews highlighted that CAS was acceptable to the AWWs and they felt that it improved their status in the community, although beneficiaries’ views were more ambivalent. Major themes highlighted include role of CAS in influencing community embeddedness of AWWs and changing the beneficiary-AWW interactions, specifically the quality of beneficiary-AWW interactions and provision of information. In this theme, we highlight how CAS was enthusiastically accepted tool for AWWs to share health information and how CAS had made AWWs service delivery more convenient. We also discussed that there were challenges in the fidelity of implementing CAS with beneficiaries offering mixed views. Physical unavailability of beneficiaries during home visits, socio-cultural norms inhibiting young married women, low levels of trust between AWWs and beneficiaries emerged as some of themes on the divergent viewpoints between AWWs and beneficiaries. Finally, technological barriers also posed a limitation to the uptake of CAS.

While the perceived ease of use of CAS and its comprehensive checklists can standardize quality of care across beneficiaries, it may also be detrimental to the quality of engagement and subsequent interactions, specifically related to the length of time spent by CHWs with beneficiaries and the amount and quality of communication, potentially influencing program fidelity. These factors should be considered and need to be addressed while implementing CAS on a large scale. This implies that use and promotion of technology or behavior change videos alone is not sufficient to engage with and counsel beneficiaries for changing health and nutrition behaviors.

Our findings from in-depth interviews demonstrate that there is a mixed perception of CAS by AWWs and beneficiaries due to the contextual reasons and the underlying challenges with the AWW-beneficiary interface. The self-reports of AWWs indicate that it had a positive perception on AWWs due to perceived improvements in their community embeddedness, standing in the community, quality of interactions, and overall service delivery. On the other hand, beneficiaries receiving services from their AWWs using CAS remained largely unaffected by the introduction of the new mHealth intervention.

Of the few beneficiaries who were exposed to CAS, most viewed CAS as a medium that improved communication and engagement. However, a few felt it rendered less personal and more rushed communication methods. Even if the videos are available in local languages creating a more visual impact, it is worth noting that videos may be viewed as a substitute to interpersonal communication between the AWWs and the beneficiaries. This may further aggravate the low levels of engagement and relationship between AWWs and beneficiaries, especially if limited verbal communication makes beneficiaries uncomfortable with asking questions, clarifying myths and sharing their personal health issues.

Many AWWs identified technological barriers to the implementation of CAS, such as early adoption problems with respect to use of CAS and technical challenges including electricity to charge their smartphones, poor battery back-up of smartphones, limited network coverage, and low internet access.

Even though the stated objectives of our study were to understand perceptions of AWWs and beneficiaries on the uptake of CAS as well as how CAS may have influenced the nature and quality of interactions, the study findings brought to fore the underlying weak linkages in the relationship between the AWWs and beneficiaries that merit further exploration. The low video viewership, the limited communication, quality of interactions and poor usage of CAS in front of the beneficiaries could be, driven by the deeper issues of AWWs community embeddedness, mistrust between AWWs and beneficiaries, and limited engagement of beneficiaries with the AWWs. The low uptake of CAS by beneficiaries could also be due to existing socio-cultural barriers or because women remain unavailable and inaccessible to AWWs when they go for home visits, and technological barriers.

There are several underlying socio-cultural challenges at the community-level that cannot be solved by technology alone. It is well-documented that gender and power dynamics do not favour women in India, especially in rural areas, affecting their social status as well as some of the reproductive health outcomes [43]. To add to this, beneficiaries work both at home and in the agricultural fields in this setting, spending time away from home. Being away from home or having busy work schedules may not allow them sufficient time to engage with their CHWs. Even when beneficiaries are at home, there is a *ghunghat* (veil or loose end of the saree or garment worn by married women to cover their head and often their face) culture effecting control on conduct of married women in domestic and familial sphere [44]. This could restrict beneficiaries from openly discussing issues with their AWWs in the presence of their in-laws and senior members at home. Further, we learned that it was common practice for beneficiaries in these regions to return to their natal home for delivery, especially for their first delivery. When beneficiaries spend a substantial part of their perinatal period in a different village than their regular residence, the continuum of care delivered by AWWs may be hampered. However, CAS could potentially offer a solution in the future to connect and refer beneficiaries to AWWs in their natal villages that could positively influence care, but this has not yet been addressed by current version of CAS.

Past research has shown the importance of trust and community-embeddedness with CHWs in India. A recent systematic review of existing reviews of CHWs found 14 reviews that highlighted the importance of community embeddedness as an enabler for success of CHW program success [45]. There is global evidence to suggest that CHWs who were embedded in their communities, higher trust and respect with their community members and a sense of ownership over the program, increased their credibility in the community. Further, social relations of trust and credibility in the community have been demonstrated to be crucial for CHWs to serve as an effective link between the health systems and their communities and provide better services to their beneficiaries [33,46,47]. The findings from our study suggest the use of mHealth applications, such as CAS, as a job aid for CHWs to reach the right beneficiary at the right time cannot be fully effective unless the CHW is enabled by strong engagement, embeddedness and standing in their communities. Further, CAS can supplement the quality of interactions with beneficiaries, husbands and in-laws that allow for better engagement with the beneficiaries, but CAS alone is not a tool to build trust and credibility among beneficiaries. Finally, there are socio-cultural norms and traditions that CHWs are aware of and need to work around. For instance, in certain communities, the recently delivered women are not allowed to freely go out or go to the AWC to pick up their food supplements from the AWC or be part of community-based events at the AWC. Although our interview guides did not have specific questions regarding women’s agency and autonomy in a household, our findings highlight that these continue to pose barriers for some AWWs to engage with directly with beneficiaries.

Our results should be interpreted in the light of limitations. First, even though data saturation for beneficiaries was achieved, beneficiary interviews were shorter in duration than expected, perhaps suggestive of the cultural norms that AWWs themselves struggle with. Even though the interviews were held in a private setting within the confines of the household, due to the nature of household set-up in specific parts of rural Madhya Pradesh and Bihar, beneficiaries may have felt discomfort to speak openly on issues due to the presence of in-laws and/or other members in the household. It is also possible that because these interviews were conducted within 6 months of the introduction of CAS, the beneficiaries had not sufficiently engaged with CAS and had not seen the AWWs use CAS in front of them, although all beneficiaries sampled should have seen an AWW in that time period according to program rollout. Another limitation could be social desirability bias, potentially evidenced by the discrepancy between what the AWWs and beneficiaries mentioned, with AWWs feeling pressured to report CAS use to outsiders (the research team) who they may have perceived as monitoring their performance.

The major strength of this qualitative study was the opportunity to understand the early experiences of AWWs and beneficiaries’ using CAS in one of the largest government-run nutrition programs in the world. To our knowledge, this is one of the first studies that comprehensively presents the complementary viewpoints of both AWWs and beneficiaries on the use of a mHealth intervention in rural India, or elsewhere in the same timeframe. As stated earlier, most mHealth intervention studies have not reported on beneficiary perceptions of the use of mHealth tools for CHWs, yet this study reveals it is important that future mHealth interventions are designed and improvised based on continuous feedback from both CHWs and beneficiaries. Assessments are necessary to evaluate if the technology is poised to adequately address socio-cultural barriers and contextual factors related to uptake of optimal maternal, neonatal and child health practices. Such assessments can also be done to identify areas for improvements and upgrades of current digital health interventions and technologies. While these findings cannot be generalized to all regions of India or other places in the world, they certainly demonstrate that mHealth interventions have complex implications for CHW-beneficiary-community interface and CHW-health system interface.

The study offers unique insights into the perceptions of the CHWs and beneficiaries of this specific mHealth intervention, the CAS. The introduction of technology as a job aid indeed has potential to help AWWs and improve some aspects of their interactions with beneficiaries. However, simply providing a mHealth tool, without addressing other social barriers, may not be effective in changing beneficiary behaviors. Additionally, we need to think critically about factors that may make things easier for AWWs, but actually decrease the quality (length, amount of personal communication) of interactions with beneficiaries. Furthermore, more research is needed to understand the reasons for the disconnect and mistrust between the AWWs and beneficiaries and the underlying mechanisms that led to this. The use of mHealth technology such as CAS can enable AWWs to improve their service delivery but cannot play a role in overcoming social and cultural barriers that have the capacity to impede improved health behaviors. Success of such an initiative depends on the acceptability, accessibility, and usability by beneficiaries, families and a strong underlying AWW-beneficiary interface and engagement.

## Supporting information

S1 File COREQ checklist. (DOC)

S1 Data Codebook. (XLSX)
